# Mammary Paget Disease With Melanocytic Proliferation Mimicking Malignant Melanoma *in situ*: A Case Report

**DOI:** 10.3389/fmed.2022.839954

**Published:** 2022-03-21

**Authors:** Shijia Rao, Alun Wang, Wei Liu, Hongling Yin, Ji Li, Lemuel Shui-Lun Tsang, Yuhui Wu, Wei Shi

**Affiliations:** ^1^Department of Dermatology, The Second Xiangya Hospital, Central South University, Changsha, China; ^2^Department of Pathology, Tulane University School of Medicine, New Orleans, LA, United States; ^3^Department of Oncology, Xiangya Hospital, Central South University, Changsha, China; ^4^Department of Pathology, Xiangya Hospital, Central South University, Changsha, China; ^5^Department of Dermatology, Xiangya Hospital, Central South University, Changsha, China; ^6^College of Medicine, The University of Tennessee Health Science Center, Memphis, TN, United States; ^7^Department of Breast Surgery, Xiangya Hospital, Central South University, Changsha, China

**Keywords:** mammary Paget disease, melanoma *in situ*, melanocytic proliferation, dermatology, pathology

## Abstract

Several cases of pigmented mammary Paget’s disease (PMPD) mimicking cutaneous malignant melanoma have been reported. In these cases, the tumor cells are colonized by melanocytes, particularly with the presence of a population of melanocytes staining for HMB-45 and S100. Here, we report a case of mammary Paget disease (MPD) which was misdiagnosed as melanoma *in situ* due to the interpretation of the staining of melanocytic markers S-100, Melan-A, and HMB-45. The tumor cells strongly expressed CK7 and GATA3, and a dual-labeling showed negative PHH3 labeling for the melanocytes. Pathologists need to be aware of the caveat of colonization of melanocytes in Paget disease.

## Introduction

Pigmented mammary Paget’s disease (PMPD) is a rare clinicopathologic variant of Paget disease, an intraductal mammary carcinoma or invasive breast carcinoma extending into the epidermis of the nipple and areola (1). The lesion of PMPD may clinically and histopathologically mimic malignant melanoma. In fact, several PMPD cases mimicking cutaneous malignant melanoma have been reported (2–5). Requena and colleagues previously summarized the histopathologic differential diagnosis between melanoma *in situ*, PMPD, and pigmented epidermotropic breast carcinoma (1) and concluded that these lesions result from the proliferation of dendritic melanocytes accompanying the intraepidermal spread of Paget cells or from melanin accumulation within the cytoplasm of intraepidermal breast carcinoma cells. Other cases have shown Paget cells being negative for Melan-A (6) while being positive for HMB-45 (7) and/or S100 (8). Here, we report a case of mammary Paget disease (MPD) with tumor cells positive for CK7, S-100, and HMB-45, and Melan-A-positive dendritic melanocytes surrounding individual and nested Paget cells raising a possibility of a collision of primary malignant melanoma and Paget disease. A dual-labeling of Melan-A and PHH3 helped to reach a diagnosis of Paget disease with melanocytic proliferation mimicking melanoma *in situ*.

## Case Report

**FIGURE 1:**
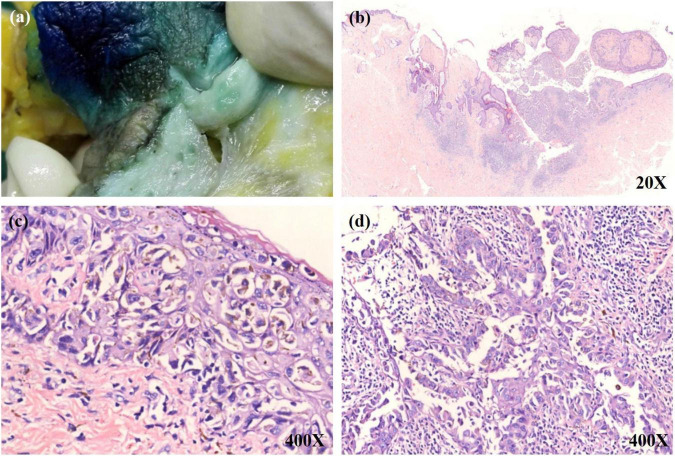
**(a)** A little bit pigmentation on the overlying epidermis of the cut surface. **(b)** A low power view shows a tumor mass in the upper dermis. The tumor nests have infiltrated into the overlying epidermis. **(c)** Numerous single and small aggregations of atypical cells with large hyperchromatic nuclei and pale-staining cytoplasm throughout the epidermis. Melanin was observed within some tumor cells. **(d)** The invasive tumor cells were colonized on lactiferous ducts of the deep dermis.

**FIGURE 2:**
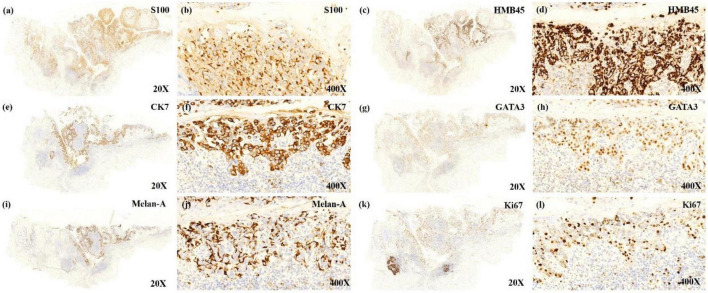
The low power view shows tumor cells expressing the immunoreactivity of S100 **(a)**, HMB45 **(c)**, CK7 **(e)**, GATA3 **(g)**, MelanA **(i)**, Ki67 **(k)**. The positively expressed cells that almost localize on the same place seem to make the diagnosis of malignant melanoma (MM) *in situ* with Paget disease to be established. The high power view shows immunoreactive cells of S100 **(b)**, HMB45 **(d)**, CK7 **(f)**, GATA3 **(h)**, and Ki67 **(l)**, indicating that these cells are Paget cells expressing S100 and HMB45. Melan-A positive expressed cells **(j)** mostly surrounded Paget cells, and the stain was mostly positive on the dendritic spine of proliferated melanocytes, implying proliferated melanocytes.

**FIGURE 3:**
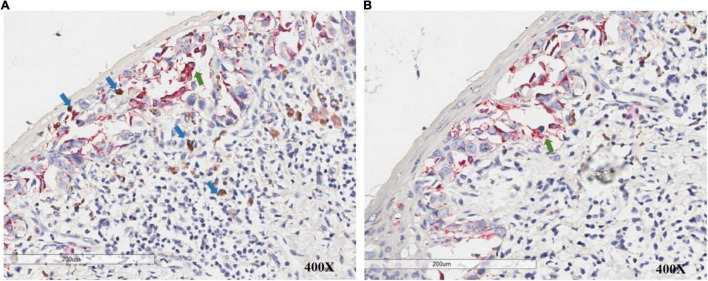
PHH3/Melan-A dual staining allows accurate discrimination of melanocytes (green arrow) and adjacent mitotic figures in non-melanocytes (blue arrow) in the intraepidermal/junctional and dermal area.

A 54-year-old Chinese woman presented with a right breast mass for 2 months. The tumor was found to be 2 cm in greatest diameter *via* ultrasound examination. Neither invagination of the nipple nor obvious color change of the overlying skin was noted. Mammography and MRI strongly suggested adenocarcinoma of the breast. Modified radical mastectomy was performed. The excised breast specimen contained a 2.0 cm × 1.8 cm × 1.8 cm mass lesion. The tumor was solid with grayish color with a small amount of pigmentation on the overlying epidermis of the cut surface (Figure 1a). Biopsy revealed an infiltrating ductal carcinoma of the breast, with tumor cells expressing cytokeratin 7 (CK7), GATA3, E-cadherin, and human epidermal growth factor receptor 2 (HER-2), while being negative for estrogen receptor (ER), progesterone receptor (PR), cytokeratin 5/6 (CK5/6), and epidermal growth factor receptor (EGFR). Additionally, approximately 30% of the tumor cells were positive for Ki-67 (Figures 2k,l). The invasive tumor cells were also found colonizing lactiferous ducts of the deep dermis (Figure 1d). The margin was clear with no involvement of vasculature or nerves. Seven sentinel lymph nodes and 10 right axillar lymph nodes were isolated, and none showed metastatic carcinoma. The tumor was staged as IIA (T2N0M0). In the overlying epidermis, a prominent pagetoid spread of either singular or nested atypical epithelial cells with abundant melanin granules was found distributing throughout the nipple epidermis (Figures 1b–d), which was highlighted on S100 and HMB45 stains (Figures 2a–d). Melanoma *in situ* was the first diagnosis. Considering Paget disease can be positive for both S100 and HMB45 (6–9). Afterward, CK7, GATA3, and Melan-A stains were added and, unexpectedly, all of these stains were positive (Figure 2e–j). Lastly, a dual-stain for Melan-A and PHH3 was carried out, and the cells expressing Melan-A showed PHH3 negativity (Figure 3). Ultimately, a diagnosis of MPD with melanocytic proliferation was reached. Currently, the patient is free of cancer without additional treatment for 5 years.

## Discussion

It is well known that both invasive and intraductal mammary carcinoma invade the epidermis of the nipple and areola, resulting in MPD. The neoplastic cells invading the epidermis are pagetoid epithelial cells, showing large, atypical, abundant pale cytoplasm. It can be difficult to differentiate MPD and malignant melanoma *in situ* (2–5). In particular, Paget cells occasionally exhibit cytoplasmic melanin granules, forming PMPD. Under the reflectance confocal microscopy, pagetoid cells (some with melanin pigment) and proliferative dendritic melanocytes could also be observed, which indicated that PMPD can mimic the feature of melanoma, increasing the risk of misdiagnosis (10). Nevertheless, histology could provide clues to differentiate these two disorders. The tumor cells in Paget disease form small aggregations and solitary unit-like epithelial cells infiltrating the basal portion of the epidermis or singly infiltrating the upper layers of epidermis. In melanoma *in situ*, however, melanocytes stack vertically in the lower half to two-thirds of the epidermis, with or without pagetoid melanocytes spreading to the granular layer. In addition, the melanocytes exhibit highly atypical, hyperchromatic nuclei with macronuclei (1). It has been proposed that melanocytic chemoattractant released from Paget cells stimulates melanocytic proliferation, and melanin pigment that is transferred from surrounding dendritic melanocytes to the tumor cells, mediated by chemotactic factors and cytokine, accounts for the occurrence of pigmentation in pigmented Paget disease (2). In tandem with histology, immunohistochemistry (IHC) can aid in differentiating Paget disease from melanoma *in situ*. Stain for S100, HMB-45, and Melan-A indicates a melanocytic tumor, while stain for CK7 yields a high diagnostic accuracy for MPD. However, IHC stains may also show inconclusive results. S100 has high sensitivity but low specificity for melanocytes due to its expression in normal breast epithelial cells, myoepithelial cells, and in both ordinary and metaplastic breast carcinomas (8). An S100 stain in Paget cells has been reported in 12 out of 20 cases of MPD (9). HMB-45 is relatively specific for melanocytes, but stain in Paget cells has also been reported (6). It has also been documented that epithelial cells with HMB-45 labeling are associated with melanocytic proliferation due to melanosomes transferring to the epithelial cells (11). Melan-A protein is a product of the MART-1 gene encoding melanocyte-associated antigen related to glycoprotein 100 (gp100). These antigen moieties are located within premelanosomes and can serve as targets for cytotoxic T lymphocytes in patients with melanoma (12). The immunostaining for melanocytes is rather sensitive and specific between those of S100 (sensitive but not specific) and HMB-45 (specific but not sensitive) (13). Besides the bodies, dendritic processes of melanocytes are also highlighted on Melan-A stain, which could lead to an overestimation of the numbers of melanocytes in the epidermis, in which dendritic processes of melanocytes could be prominent. This overestimation could lead to an impression of melanoma *in situ*. El Shabrawi-Caelen et al. (12) found that non-specific MART-1/Melan-A staining can occur in sun-damaged skin. They speculated that MART-1 may stain keratinocytes and other non-melanocytic cells which were damaged by inflammation. Maize et al. (14) described a patient with discoid lupus erythematosus (DLE) pigmented lesions who was misdiagnosed to have a regressed melanocytic lesion. It was based upon a finding that Melan-A positive cell nests located in the dermal-epidermal junction that, upon further testing, failed to stain for S100 or a “melanoma cocktail” of tyrosinase and HMB-45.

Primary melanoma on the nipple is rare, especially in female. To our knowledge, only a few cases have been documented (15). Padmore et al. reported two cases of primary combined malignant melanoma and ductal carcinoma of the breast in 1996 (16). Using double-labeling IHC, they found that the epithelial portion was cytokeratin positive, the melanoma portion was HMB-45-positive, and both portions were S-100-positive. Due to the intimate admixture of neoplastic ductal and malignant melanoma cells present in a single tumor mass, the authors speculated that these lesions have a single tumor of breast origin with bidirectional differentiation.

Regarding the current case, under a low-power view, significant overlap was found between cells positive for CK7, GATA3, HMB45, Melan-A, and S100. However, with careful observation under higher powers, we found that the Melan-A-positive cells were mostly surrounded by CK7-positive cells, and the stain was mostly positive on the dendritic spine of proliferated melanocytes (Figures 2e,f,i,j). PHH3 is a core histone protein and is present only during mitosis. Particularly, the PHH3 stain highlights mitotic figures from early prophase through metaphase, anaphase, and telophase. PHH3/Melan-A dual staining could help to accurately identify mitotic figures in melanocytes versus non-melanocytes (17). We further found that there were no PHH3 positive cells among Melan-A positive cells on dual staining, which indicated that the melanocytes were not undergoing mitosis. For this case, we speculate that the inflammatory process of the Paget cells stimulated melanocytic and dendritic spine proliferation, while specific GP-100 melanosomes produced by proliferated melanocytes were carried into the cytoplasm of Paget cells. Melanocytic chemoattractant was released from Paget cells, which increased the numbers of dendritic melanocytes and melanophages. This sequence of events stimulated melanocyte proliferation which would play a role in generating pigmentation of this lesion, mimicking malignant melanoma.

In conclusion, as the overlap features between PMPD and melanoma *in situ* are clinically and pathologically common, using a panel of IHC with detailed analysis would be essential for reaching a correct diagnosis. A dual-labeling of Melan-A and PHH3 could be helpful.

## Data Availability Statement

The raw data supporting the conclusions of this article will be made available by the authors, without undue reservation.

## Ethics Statement

The patient in this manuscript has given written informed consent to the publication of her case details.

## Author Contributions

WS was responsible for planning this work. AW made a dual stain of Melan-A and PHH3 in the Department of Pathology, Tulane University and confirmed the final diagnosis. SR was responsible for drafting this manuscript. HY and WL were responsible for providing professional opinions from Departments of Pathology and Oncology, respectively. WS, AW, JL, LS-LT, and YW were responsible for revising this manuscript. YW and WS approved the final manuscript. All authors contributed to the article and approved the submitted version.

## Conflict of Interest

The authors declare that the research was conducted in the absence of any commercial or financial relationships that could be construed as a potential conflict of interest.

## Publisher’s Note

All claims expressed in this article are solely those of the authors and do not necessarily represent those of their affiliated organizations, or those of the publisher, the editors and the reviewers. Any product that may be evaluated in this article, or claim that may be made by its manufacturer, is not guaranteed or endorsed by the publisher.

## Supplementary Material

Supplementary Figure 1 Paget cells surrounded by dendritic melanocytes.
