# Supplementary figures and images for: Mammary Paget Disease With Melanocytic Proliferation Mimicking Malignant Melanoma in situ: A Case Report
Source: Front Med (Lausanne). 2022 Mar 21;9:839954. doi: 10.3389/fmed.2022.839954 (PMC8978827; doi:10.3389/fmed.2022.839954)

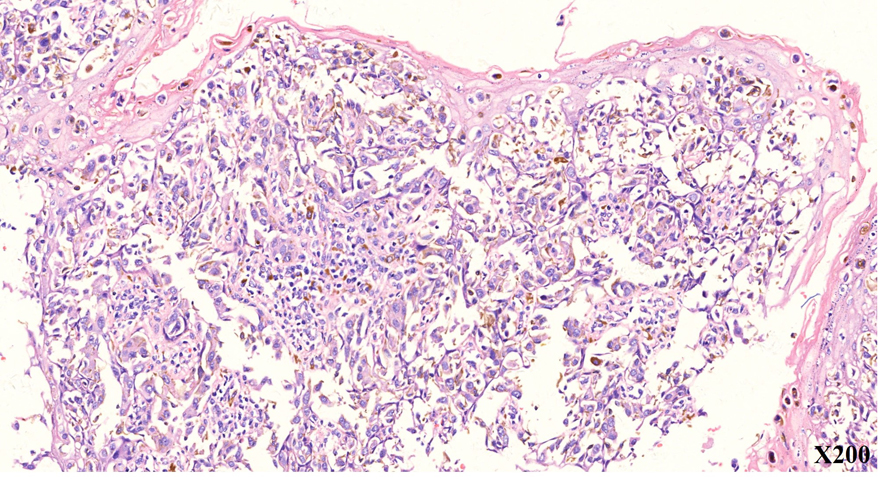

Supplement: Supplementary Figure 1 — Paget cells surrounded by dendritic melanocytes. [file Image_1.JPEG]
